# Lock out: targeting TMPRSS2 to block influenza and coronaviruses

**DOI:** 10.1128/jvi.00807-25

**Published:** 2026-05-04

**Authors:** Lu Zhang, Markus Hoffmann, Stefan Pöhlmann

**Affiliations:** 1 Infection Biology Unit, German Primate Center – Leibniz Institute for Primate Research 28361, Göttingen, Germany; 2 Faculty of Biology and Psychology, Georg-August-University Göttingen 98900, Göttingen, Germany; Universiteit Gent, Merelbeke, Belgium

**Keywords:** TMPRSS2, protease, spike, SARS-CoV-2, priming

## Abstract

Coronaviruses and influenza A viruses (IAV) can cause severe respiratory disease and have pandemic potential. Both viruses depend on priming of their glycoproteins by host cell proteases for the acquisition of infectivity, and the responsible enzymes represent potential targets for intervention. Initial studies suggested that these viruses may exploit redundant proteolytic systems. However, research conducted over the last two decades has pointed to a key role for a single enzyme in coronavirus and IAV priming, the transmembrane protease serine 2 (TMPRSS2). Interest in TMPRSS2 as a host dependency factor and therapeutic target intensified during the COVID-19 pandemic, prompting extensive investigation into its biology, substrate specificity, and pharmacological inhibition. Here, we review recent efforts to define the role of TMPRSS2 in coronavirus infection and to target this protease for antiviral intervention.

## INTRODUCTION

The COVID-19 pandemic has highlighted the vulnerability of health systems and economies to the rapid global spread of novel, pathogenic respiratory viruses. Despite significant efforts to repurpose known drugs and to devise novel ones to combat SARS-CoV-2, limited therapeutic options are available. Since these therapeutics target viral enzymes, the main protease (1) and the polymerase (2, 3), resistance development can compromise therapeutic efficacy, and the breadth of antiviral activity is limited. Targeting host cell factors rather than virus-encoded factors offers the prospect of a high genetic barrier to resistance development, owing to the greater genetic stability of host factors relative to viral factors. In addition, this strategy may confer broad antiviral activity if the targeted host factors are exploited by diverse viruses for their replication and spread. However, the results of genome-wide RNAi or CRISPR screens for host cell factors can be challenging to interpret (4), and unwanted toxic effects might be associated with targeting certain factors, complicating this approach.

It was discovered in the 1970s that influenza A viruses (IAVs) critically depend on cellular proteases for the acquisition of infectivity (5). These enzymes cleave the viral hemagglutinin (HA) protein, which mediates viral entry into cells, thereby converting it from an inactive into an active form in a process termed priming. However, subsequent studies revealed that besides trypsin, an intestinal serine protease used to support viral spread in cell culture, many other cellular proteases can also prime HA (see reference 6 for an example). These findings suggested that IAV can exploit redundant proteolytic systems for HA priming, which would make intervention challenging. However, more recent studies in cell cultures and mice provided evidence that a single enzyme, the transmembrane protease serine 2 (TMPRSS2), a member of the type II transmembrane serine protease (TTSP) family, has a key role in priming and infectivity of several IAV (7–9). In parallel, it was discovered that other respiratory viruses, including coronaviruses, parainfluenza viruses, and human metapneumoviruses (10–16), and a non-respiratory virus, hepatitis C virus (17), can also exploit TMPRSS2 for priming in cell culture, suggesting that TMPRSS2 inhibitors might exert broad antiviral activity.

The finding that SARS-CoV-2, like SARS-CoV, depends on TMPRSS2 for spike (S) protein cleavage and entry into Calu-3 lung cancer cells (18), reported early in the COVID-19 pandemic, triggered multiple efforts to obtain insights into the proteolytic processes underlying TMPRSS2-dependent cell entry, to solve the TMPRSS2 structure and to transform this knowledge into candidate antivirals, several of which were tested in clinical studies. Here, we will review progress in these areas, with a focus on coronaviruses.

## STRUCTURE AND EXPRESSION OF TMPRSS2

**Fig 1:**
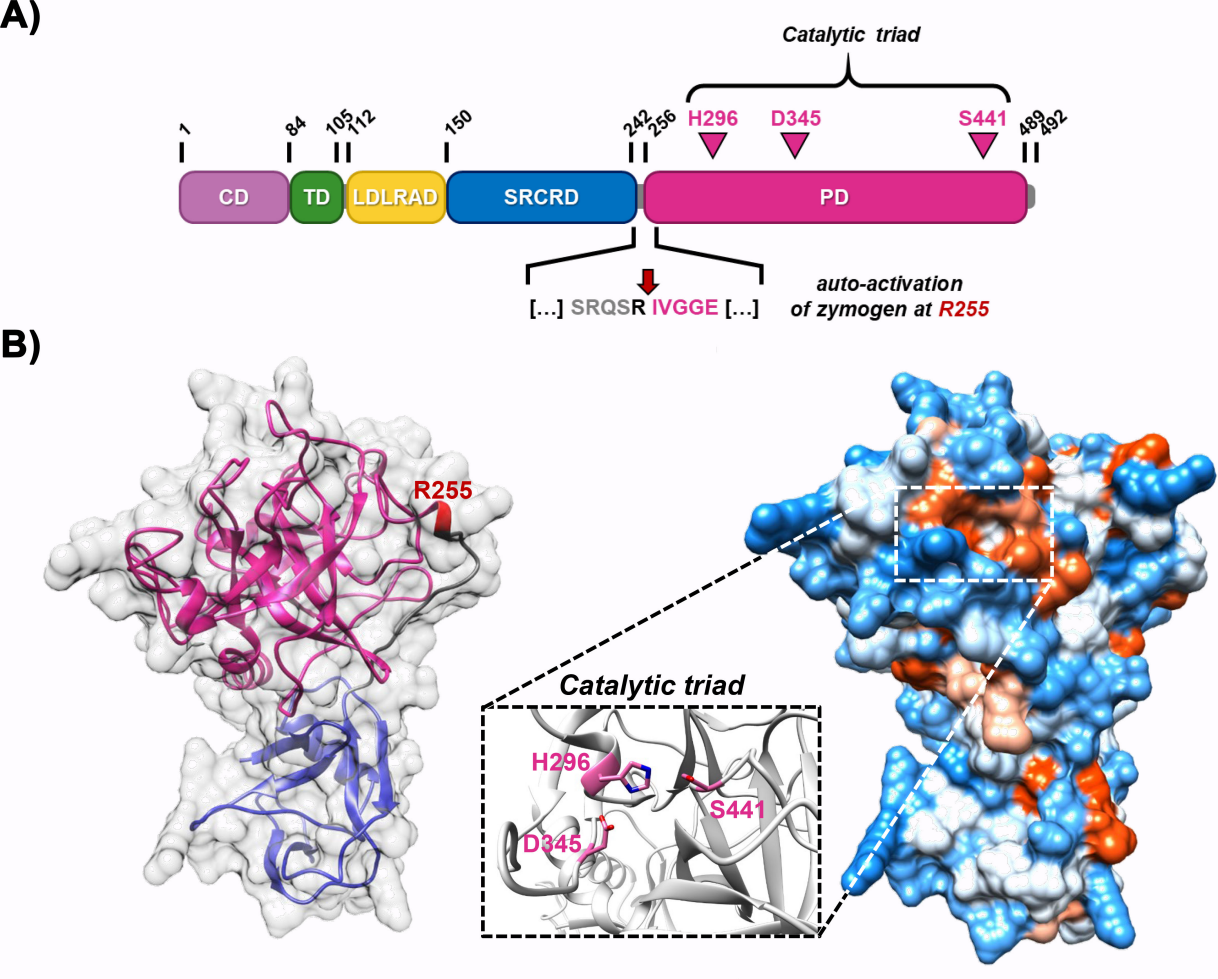
Structural organization of TMPRSS2. (**A**) Domain organization of the zymogen form of the type-II transmembrane serine protease TMPRSS2, in which the cleavage site used for auto-activation, which is located upstream of the protease domain, and the three amino acid residues forming the catalytic triad of the catalytic site are indicated. CD, cytoplasmic domain; TD, transmembrane domain; LDLRAD, low-density lipoprotein receptor class A domain; SRCRD, scavenger receptor cysteine-rich domain; PD, protease domain. (**B**) Left: Protein structure of TMPRSS2 in which the SRCRD (blue) and PD (pink) are highlighted. Further, the arginine residue at position 255, which represents the cleavage site for auto-activation of the zymogen form, is indicated. Right: Protein structure of TMPRSS2 in which hydrophobic (red) and hydrophilic (blue) amino acid residues are indicated. The cleft harboring the enzymatic site with the catalytic triad (His296, Asp345, and Ser441) is indicated. The protein models are based on the crystal structure PDB:7MEQ (19).

TMPRSS2 exhibits the following domain organization: the N-terminal cytoplasmic tail is followed by a transmembrane domain and, extracellularly, by a low-density lipoprotein-receptor class A (LDLR-A) domain, a scavenger-receptor cysteine-rich (SRCR) domain and a C-terminal trypsin-like serine protease domain (19). The structure of the SRCR and the protease domain in complex with the inhibitor Nafamostat has been resolved at 1.95 Å (19). The protease domain contains a catalytic triad, His296, Asp345, and Ser441, and is the central target for inhibitors. It is activated by autoproteolytic (self-) cleavage between Arg255 and Ile256, which may result in the secretion of the protease domain (20) (Fig. 1). Autoproteolytic activation enables TMPRSS2 to hydrolyze protein substrates at appropriate cleavage sites, with cleavage typically occurring after single lysine or arginine residues (21). Proteolysis commences with nucleophilic attack by Ser441, generating a tetrahedral intermediate that collapses into an acyl-enzyme intermediate. The catalytic process is facilitated by His296, which is stabilized by Asp345 and mediates proton transfer during both acylation and deacylation, after which the acyl-enzyme intermediate is hydrolyzed (22, 23). The protease domain exhibits a chymotrypsin-like/trypsin-like fold that is highly conserved among TTSPs, including the catalytic center, and substrate specificity of TTSPs is mainly conferred by surface-exposed loops (19). In addition, a highly conserved, unpaired cysteine residue (Cys379), which is either absent in other TTSPs or involved in disulfide bond formation, may contribute to ligand recognition (19). The structure of the SRCR domain of TMPRSS2 is similar to that of TMPRSS13 and might play an important role in ligand recognition by orienting the protease domain in space (19).

TMPRSS2 is expressed at significant levels in respiratory, gastrointestinal and, most prominently, prostate epithelia (24, 25) but was also detected in other tissues, and its promoter contains an androgen-responsive element (ARE). In prostate cancer, a fusion of the *TMPRSS2* and *ERG* genes can drive cancer development because of androgen-driven overexpression of the oncogenic ERG under the control of the TMPRSS2 promoter (26). In contrast, TMPRSS2 was reported to be dispensable for homeostasis and development (27), and its biological function has not been fully defined, although recent studies point toward a role in innate responses and maintenance of the epithelial barrier, as discussed below.

## TMPRSS2 AS THERAPEUTIC TARGET—EVIDENCE AND CHALLENGES

**Fig 2:**
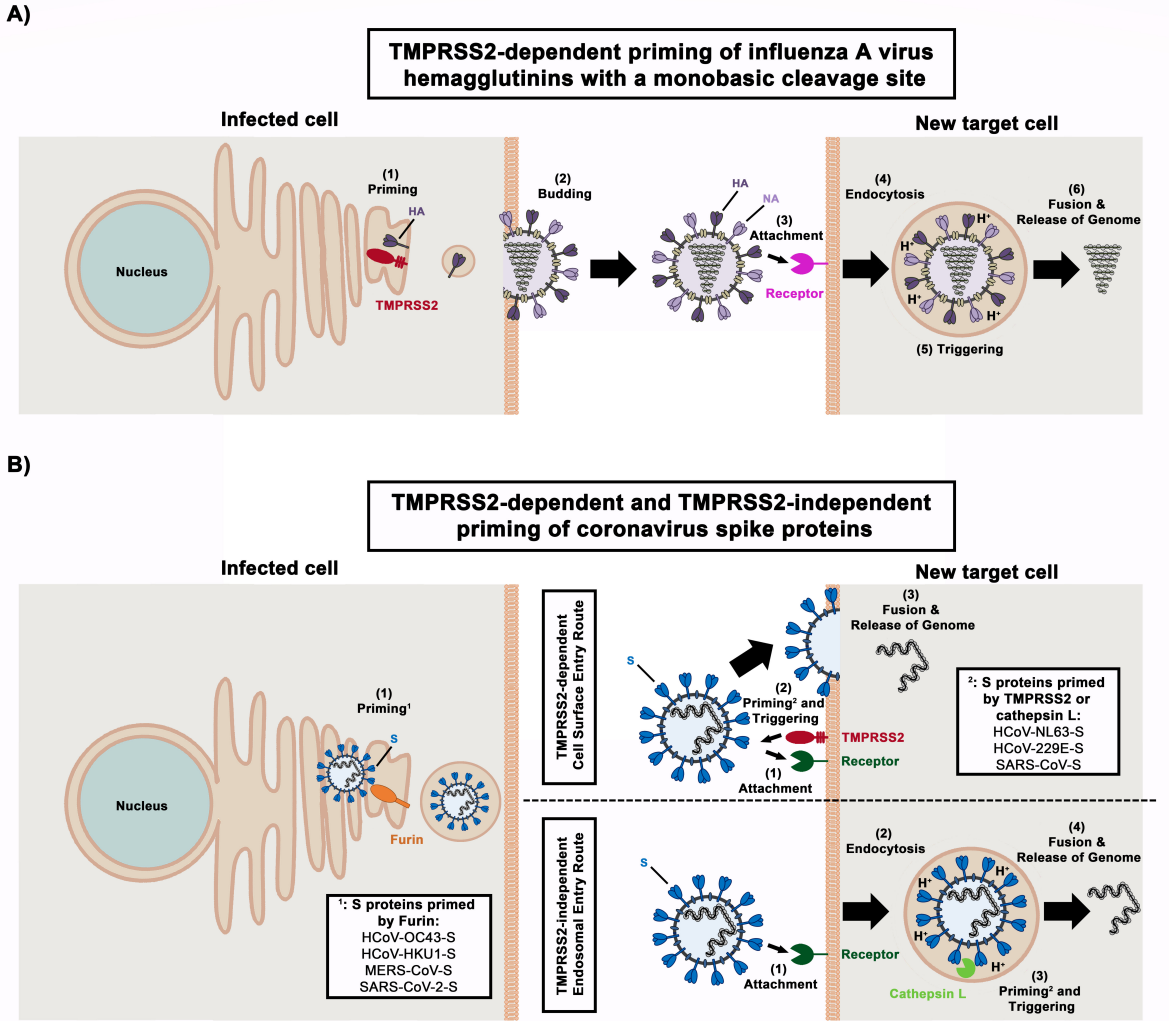
Priming of coronavirus spike proteins and influenza A virus hemagglutinins (HAs). (**A**) TMPRSS2-mediated priming of influenza A virus hemagglutinins (HA) with monobasic cleavage sites in the trans-Golgi network of infected cells enables subsequent HA-driven cell entry into new target cells, where HA-mediated fusion is triggered by endosomal low pH. (**B**) Left: Priming of certain coronavirus spike (S) proteins (HCoV-OC43-S, HCoV-HKU1-S, MERS-CoV-S, and SARS-CoV-2-S) by furin in the trans-Golgi network of infected cells. Top right: TMPRSS2-mediated priming and triggering of coronavirus S proteins after attachment to the respective cellular receptor on target cells leads to S protein-mediated fusion at the plasma membrane. Bottom right: In the absence of TMPRSS2, an auxiliary entry route is employed in which S proteins are primed and triggered by cathepsin L in endosomal vesicles following particle internalization, leading to S protein-mediated fusion at the endosomal membrane. HA, hemagglutinin; NA, neuraminidase; S, spike protein.

Priming of IAV hemagglutinin (HA) requires a single proteolytic cleavage at the boundary between the receptor-binding subunit HA1 and the membrane-anchored, fusogenic subunit HA2. This cleavage occurs during viral maturation in infected cells and renders HA membrane fusion-competent, allowing the conformational changes required for membrane fusion to be triggered by low pH following viral uptake into late endosomes (Fig. 2). In contrast, the coronavirus S protein requires two successive cleavage steps for priming and subsequent membrane fusion. The first occurs at the S1/S2 site located at the border between the surface unit, S1, and the transmembrane unit, S2. The second occurs at the S2′ site located within the S2 subunit, and it is believed that this cleavage step already triggers or at least accelerates S protein-driven membrane fusion (28). Thus, priming and triggering are coupled for coronavirus S proteins and will be collectively referred to as proteolytic activation for the purpose of this discussion. Importantly, coronavirus S proteins can employ several proteases for proteolytic activation, and priming and triggering can occur at different cellular locations. For entry into cell lines, coronaviruses frequently employ the endosomal cysteine protease cathepsin L, which cleaves the S protein at the S2′ site. In addition, coronaviruses can engage the cell surface protease TMPRSS2 for entry (Fig. 2), which is expressed only in a few cell lines, including Calu-3 lung cancer and Caco-2 colon carcinoma cells. TMPRSS2 is essential for robust SARS-CoV (10–12), SARS-CoV-2 (18, 29), and MERS-CoV (30, 31) entry into lung cells and entry requires cleavage of the S protein at the S2′ site by TMPRSS2. Further, in the case of SARS-CoV-2 and MERS-CoV, lung cell entry depends on S protein priming at the S1/S2 site by furin in the trans-Golgi network of infected cells (29, 32–35). Finally, it should be noted that variations to this theme have been proposed. Thus, furin-related proteases might contribute to S protein cleavage at the S1/S2 site (36), evidence has been provided that furin might cleave the S protein at the S2′ site (37) and a TMPRSS2 and cathepsin L-independent, metalloproteinase-dependent entry pathways have been documented (38). However, it is largely unknown whether these processes contribute to viral spread in the infected host.

The availability of an auxiliary, cathepsin L-dependent entry pathway (18, 39), the finding that several TTSPs can prime SARS-CoV-2 S protein upon overexpression and are endogenously expressed in viral target cells in the respiratory tract (24, 40–42), as well as the marked cathepsin L-dependence of SARS-CoV-2 Omicron variants for entry into cell lines (43–45), raised the question of whether TMPRSS2 activity is essential for SARS-CoV-2 spread and constitutes a valid target for antiviral intervention in the context of coronavirus infection. In the following, we will discuss progress and open questions in this area.

### Impact of TMPRSS2 polymorphisms on influenza and COVID-19 development

One way to address whether TMPRSS2 is important for viral spread is the analysis of polymorphisms in the TMPRSS2 gene, which may modulate expression and/or enzymatic activity of the protein and may thus impact susceptibility to infection and disease. Evidence for such an association was first described in the context of IAV infection. Cheng et al. identified the GG genotype of rs2070788 to be associated with increased TMPRSS2 expression and augmented risk for severe influenza upon infection with the H1N1 IAV responsible for the influenza pandemic in 2009 (46). rs383510 was found to be in linkage disequilibrium with rs2070788 in Asian populations (i.e., the variants are commonly co-inherited) and likely contributes to increased TMPRSS2 expression, being located in a putative enhancer region and modulating enhancer activity. Finally, both rs2070788 G and rs383510 T were associated with susceptibility to influenza upon H7N9 infection (46). However, similar results were not documented for COVID-19, and the linkage disequilibrium was not observed in several other populations (47).

In the context of COVID-19, polymorphisms reducing TMPRSS2 expression and/or activity were shown to modify the risk for disease development. *TMPRSS2* (21q22.3) was found to be associated with COVID-19 outcome. The lead variant: rs9305744:G>A, located in a *TMPRSS2* intron, was classified as protective against critical illness and to be in linkage disequilibrium with the *TMPRSS2* missense variant rs12329760:C>T (p.Val197Met in TMPRSS2 isoform 1 or p.Val160Met in TMPRSS2 isoform 2) (48). The Val160Met mutation is located in the SRCR domain and is associated with protection from infection and disease (49–52). Structural modeling and molecular dynamics simulations suggest that Val160Met, although distal to the catalytic triad (His296-Asp345-Ser441), alters local folding stability and shifts TMPRSS2 toward a zymogen-like, uncleaved conformation (51, 53, 54). In cell-based assays, Val160Met exhibited reduced autocatalytic cleavage, resulting in a higher proportion of membrane-bound but non-activated TMPRSS2 (51, 52). Consequently, Val160Met diminished S protein-driven syncytium formation and viral entry into cells (51, 52), although the latter effect was only observed in the context of a mutation that abrogates autocatalytic activation of TMPRSS2 (51). These findings support a model in which Val160Met reduces SARS-CoV-2 entry primarily by attenuating TMPRSS2 protease activity rather than lowering expression levels, in line with its association with milder COVID-19 in several cohorts.

### TMPRSS2 is essential for coronavirus and influenza A virus infection of mice

Knock-out (KO) mouse models provided further evidence for an important role of TMPRSS2 in IAV and coronavirus infection, as discussed below. Notably, an initial study reported that *Tmprss2* KO in mice might not be associated with a readily detectable phenotype in the absence of infection (27). Therefore, it has been suggested that other members of the TTSP family can take over the physiological functions of TMPRSS2 or that TMPRSS2 might have a non-vital function that is apparent only under certain circumstances, such as stress or disease (27). However, more recent work points toward several TMPRSS2 functions: plasmacytoid dendritic cells generated from *Tmprss2* KO animals showed reduced cytokine expression upon TLR stimulation, while the reverse effects were detected for myeloid-derived dendritic cells (55). In keeping with these findings, inflammatory chemokine and cytokine responses upon stimulation of TLR3 were attenuated in *Tmprss2* KO mice (56). Further, TMPRSS2 contributed to the activation of the epithelial sodium channel (ENaC) and was required for normal expression of the epithelial cell adhesion molecule EpCAM and the tight junction proteins claudin-3 and claudin-7 (57, 58). Thus, TMPRSS2 expression may contribute to innate responses to infection and to the maintenance of epithelial barrier integrity as well as trans-epithelial sodium transport. However, it remains unclear whether the absence of these functions contributed to the pronounced phenotype observed in *Tmprss2* KO mice upon IAV and coronavirus infection, which is discussed in the following paragraph.

#### Tmprss2 KO in SARS, COVID-19, and MERS models

Angiotensin-converting enzyme 2 (ACE2), the cellular receptor of SARS-CoV and SARS-CoV-2 (18, 59, 60), and TMPRSS2 are coexpressed in lung type II pneumocytes, enterocytes, and nasal goblet secretory cells (61) and expression increases with age (25, 62, 63). Loss of *Tmprss2* protected animals from weight loss, lung inflammation, and pathogenesis upon intranasal challenge with SARS-CoV (56). Similarly, two studies revealed that mice lacking TMPRSS2 do not suffer weight loss and lung damage upon infection with pre-Omicron variants and exhibit markedly reduced viral spread in the upper and lower respiratory tract upon infection with pre-Omicron and Omicron variants (64, 65). The dependence of Omicron subvariants on TMPRSS2 for spread *in vivo* is noteworthy, although it was not as pronounced as that observed for pre-Omicron variants, partially reflecting the preference of Omicron variants for cathepsin L over TMPRSS2 in cell culture (43–45). The role of TMPRSS2 in MERS-CoV infection was examined using mice engineered to express human DPP4, the receptor used by MERS-CoV for cell entry (66), in a WT and *Tmprss2* KO context. The absence of TMPRSS2 was protective against weight loss, high viral titers, and severe lung pathology (56), indicating that TMPRSS2 is essential for robust infection by sarbecoviruses and merbecoviruses. Although these results point toward an important role of TMPRSS2 in murine models, some studies suggest a significant contribution of cathepsin L (39), for instance, CRISPR/Cas9-mediated knockdown was associated with reduced viral load in the lung and diminished expression of proinflammatory cytokines and chemokines (67).

#### Tmprss2 KO in influenza models

The absence of TMPRSS2 markedly diminished viral spread, weight loss, disease pathogenesis, and fatal outcome upon infection with H1N1 and H7N9 (7–9) as well as IAV bearing H2 or H10 HA (68, 69). Further, HA cleavage was reduced or absent in infected animals (9), indicating that attenuation was due to lack of HA priming. *Tmprss2* KO mice remained susceptible to lethal infection by highly pathogenic avian IAV, like H5N1, which depend on furin and not TMPRSS2 for HA priming (8). Some H3N2 IAV strains showed little TMPRSS2 dependence (7, 9), causing lethal disease in *Tmprss2* KO mice (7). TMPRSS2 independence was linked to sequences in the HA surface unit HA1 (70) and required the expression of TMPRSS4 (71), which cleaved HA in cell culture and supported viral spread in cultured type II alveolar epithelial cells (72). Conversely, an H3N2 strain that was dependent on TMPRSS2 for efficient spread in mice lost TMPRSS2 dependence during passaging in *Tmprss2* KO mice, and this phenotype was associated with loss of a sequon in HA2 (73). However, the mechanism ensuring HA priming in the absence of TMPRSS2 was not elucidated. Finally, influenza B viruses (IBV) can use a broader panel of TTSPs for priming in cell culture as compared to IAV (74). As a consequence, TMPRSS2 knockdown has a less pronounced effect on IBV infection of cultured respiratory epithelial cells (74, 75), and *Tmprss2* KO is compatible with robust IBV spread and pathogenesis in mice (76).

Collectively, human pathogenic sarbecoviruses and merbecoviruses as well as several IAV rely on TMPRSS2 for robust spread and pathogenesis in rodents. However, IAV can acquire TMPRSS2 independence under selective pressure, and cathepsin L may support some level of SARS-CoV-2 spread, for instance because of upregulation of this protease upon infection (39). Further, it is noteworthy that the absence of TMPRSS2 in pigs had no impact on lung viral loads upon experimental infection with 2009 H1N1 IAV, a virus that depends on TMPRSS2 in mice, but reduced lung lesions and expression of proinflammatory cytokines (77). Thus, TMPRSS2 dependence of IAV might be species specific with TMPRSS2 promoting inflammation but not viral replication in pigs. The continued development of animal models is needed, considering that murine and human TMPRSS2 only exhibit 82% amino acid identity and differ in breadth of expression (78). Similarly, one can speculate that mice and humans differ in the expression and/or activity of natural TMPRSS2 inhibitors that may impact viral infection, including SERPINE1 (79). Studies with non-human primates might allow addressing these limitations. Finally, it needs to be determined whether viruses that are able to use TMPRSS2 in cell lines, including human metapneumovirus and parainfluenza viruses, also depend on this protease for spread in the host.

### Antiviral activity of TMPRSS2 inhibitors in cell culture and rodent models

#### Cell culture models

Several studies found that serine protease inhibitors active against TMPRSS2 and related proteases inhibit SARS-CoV-2 infection of Calu-3 lung cancer cells (29, 80), primary or stem cell-derived respiratory epithelial cells (81), precision cut lung slices (24), and organoids mimicking the upper or lower respiratory tract (82–84), while inhibitors targeting cathepsin L were less or not effective (81, 84). Similarly, knock-out of *TMPRSS2* markedly reduced infection of Calu-3 lung cancer cells (85–87), primary or stem cell-derived respiratory epithelial cells (88), and airway (82) or intestinal (82, 89, 90) organoids, while knock-out of *cathepsin L* had no or only a moderate effect (87–89). Importantly, the dependence on serine protease/TMPRSS2 activity was observed for pre-Omicron and Omicron variants, in keeping with the markedly reduced spread of Omicron variants in *Tmprss2* KO mice. However, these effects were not universally observed; one study reported a minor antiviral effect of *TMPRSS2* KO in human respiratory organoids, although entry of pre-Omicron and Omicron variants remained sensitive to a serine protease inhibitor (91), suggesting a potential role for TMPRSS2-related TTSPs, which deserves further investigation.

#### Rodent models

**Fig 3:**
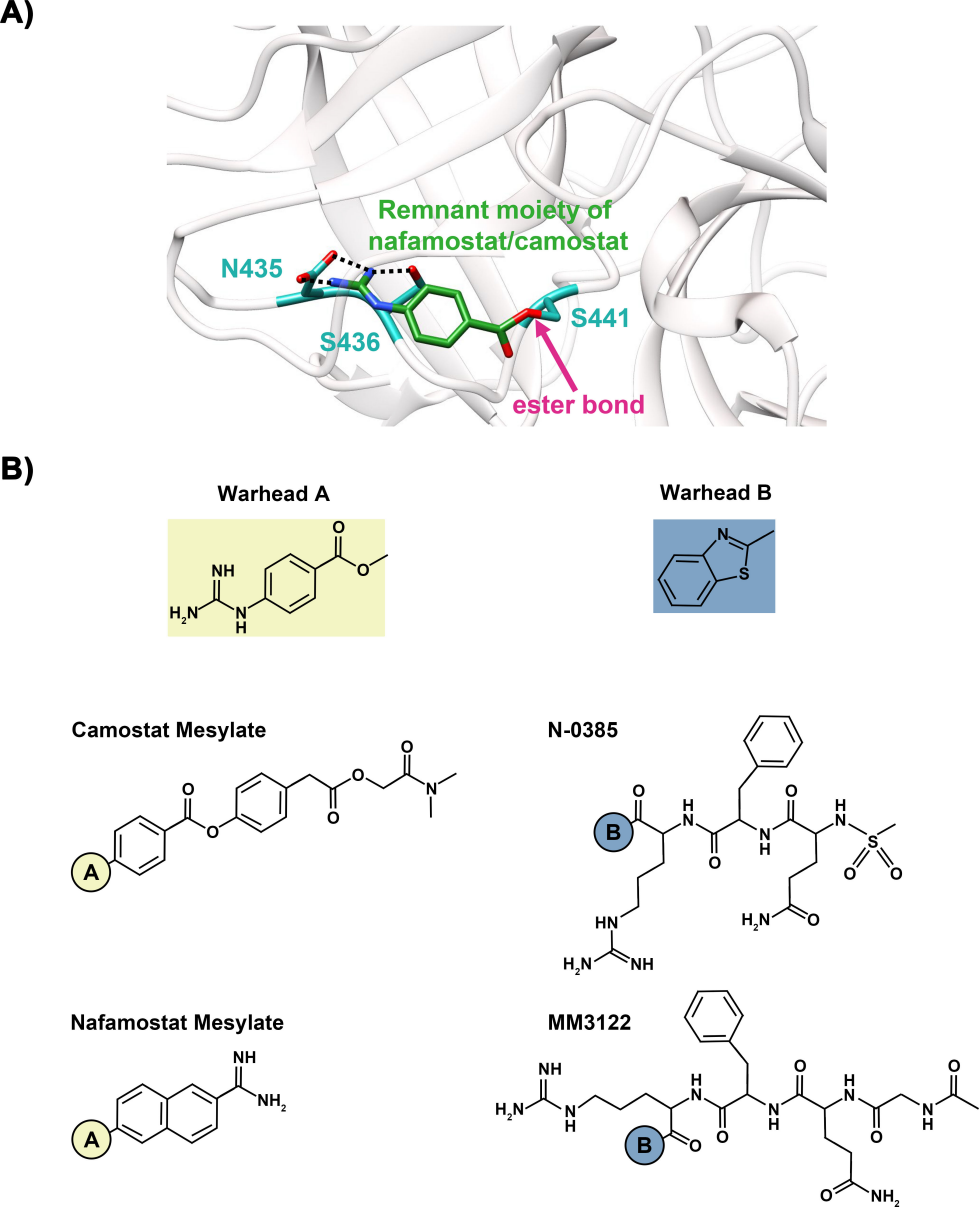
Inhibition of TMPRSS2 activity by serine protease inhibitors. (**A**) Protein model of the catalytic site of TMPRSS2 with the remnant moiety of the serine protease inhibitor Camostat mesylate/Nafamostat mesylate (green) covalently bound to residue Ser441 of the catalytic triad by an ester bond. Additional structural interactions between the remnant moiety and TMPRSS2 residues by salt bridges and hydrogen bonds are indicated by dashed lines. The protein model is based on the crystal structure PDB:7MEQ (19). (**B**) Chemical structures of the serine protease inhibitors Camostat mesylate and Nafamostat mesylate (left) as well as the specific TMPRSS2 inhibitors N-0385 and MM3122 (right).

Several of the TMPRSS2 inhibitors that showed antiviral activity in cell culture were subsequently examined in rodent models. These compounds included Camostat mesylate and Nafamostat mesylate, which are approved for treatment of chronic pancreatitis (Camostat) and acute pancreatitis/disseminated intravascular coagulation (DIC) (Nafamostat) in certain countries. They bind TMPRSS2 covalently and irreversibly (92, 93) (Fig. 3). Further, ketobenzothiazole-based inhibitors, including N-0385 and MM3122, were tested (94, 95). Both bind TMPRSS2 covalently and reversibly. Finally, other inhibitors with less defined inhibitory mechanisms have been examined, for example, the kallikrein inhibitor Avoralstat (96).

Camostat, but not a cathepsin L inhibitor, was effective in a SARS model when administered orally (97). In a separate study, Camostat also exhibited moderate antiviral activity against SARS-CoV-2 when administered via the intraperitoneal route (96). Further, potent anti-SARS-CoV-2 activity was measured for N-0385 upon intranasal application (94), and a derivative exhibited increased antiviral activity in cell culture (98). Finally, both MM3122 (99) and Avoralstat (96) were effective upon intraperitoneal application. Collectively, these results show that serine protease inhibitors active against TMPRSS2 could be suitable for COVID-19 treatment and prevention. Further, the finding that intranasal application of Nafamostat increased antiviral effects as compared to intraperitoneal application (100) has important implications for future studies. It suggests that topical application might result in higher compound concentrations in the respiratory tract than other routes of application, in agreement with findings reported by a separate study (101), allowing for more effective prevention (102) and therapy (100).

### Efficacy of TMPRSS2 inhibitors against COVID-19 in clinical studies

Since Camostat and Nafamostat were already approved for human use for unrelated diseases, several clinical trials focused on the repurposing of these compounds for COVID-19 treatment. Camostat is administered orally and is a less potent inhibitor of SARS-CoV-2 infection in cell culture than Nafamostat (103). Nafamostat has a very short plasma half-life (104) and is applied via continuous infusion. Five randomized clinical trials have shown that Camostat is safe but has no or very limited clinical benefit in the context of COVID-19 (105–109), although a shorter time to clinical improvement and reduced risk of death were observed in a sixth trial (110). No severe adverse effects were detected upon Nafamostat treatment, although in some trials, hyperkalemia, phlebitis, and bleeding were observed in a fraction of treated patients. Regarding efficacy, mixed results were reported. One trial found that treatment reduced viral loads in early-onset COVID-19 patients (111) while another study reported a 93% posterior probability that Nafamostat diminished the odds of death (112). Another found that a combination of Nafamostat and Favipiravir did not impact clinical progression but resulted in earlier defervescence and recovery of oxygen saturation (113). Finally, other studies found a shorter median time to clinical improvement in a small group of high-risk COVID-19 patients who needed oxygen treatment (114) or observed no evidence for anti-inflammatory, anticoagulant, or antiviral activity of Nafamostat (115). Collectively, clinical examination of Camostat and Nafamostat yielded largely disappointing results, with few exceptions, likely due to low compound concentrations attained in the respiratory epithelium.

Although the clinical trials discussed above failed to provide clear evidence supporting the use of Nafamostat or Camostat for the treatment of COVID-19, such evidence has been obtained for another inhibitor of trypsin-like serine proteases with activity against TMPRSS2, Aprotinin. Aprotinin inhibits serine proteases by binding to the active site in a non-covalent and reversible manner, and the spectrum of proteases inhibited by Aprotinin is somewhat narrower than that of Nafamostat (116, 117). Notably, aprotinin has been shown to block IAV infection in cell culture, animal models, and humans (118). Inhaled Aprotinin has been approved for influenza treatment in Russia based on a clinical trial showing that Aprotinin inhalation was safe and resulted in a marked reduction in viral load and disease duration (119). In the context of COVID-19, evidence was provided that Aprotinin inhibits SARS-CoV-2 infection in cell culture (120–122) and, in combination with other drugs, in rodents and humans (123, 124). More notably, in a double-blind, randomized trial comparing standard treatment versus standard treatment plus aprotinin, inhaled Aprotinin (11-day treatment, 2,000 KIU [kallikrein inhibitor units] per day) reduced the length of hospital stay by 5 days and decreased the need for oxygen therapy, with no adverse reactions observed (125). Although confirmation in independent studies and analysis of additional parameters, particularly viral load, is required, these results highlight the potential of TMPRSS2 inhibitors for the treatment of COVID-19.

Collectively, knock-down and knock-out studies both in cell culture and in mice indicate that TMPRSS2 is of central importance to IAV and coronavirus spread in the respiratory tract, although its role in the invasion of other organs is less clear. Serine protease inhibitors active against TMPRSS2 inhibit coronavirus and IAV infection in cell culture and rodent models, but topical application is needed for high antiviral activity. Unfortunately, clinical trials addressing whether Camostat and Nafamostat can be repurposed for COVID-19 treatment did not encompass topical application and provided little encouraging results. In contrast, inhaled Aprotinin was effective and safe and deserves further evaluation.

### Future directions for improving TMPRSS2 inhibitors

Future efforts should focus on topically applied inhibitors with increased efficacy and specificity as compared to present compounds. These efforts benefit from insights into the structure of the TMPRSS2 ectodomain in complex with Nafamostat, which significantly aids the understanding of substrate and inhibitor binding to TMPRSS2 (19). Structure-based insights into TMPRSS2 substrate recognition and its inhibition have also been obtained from studies of TMPRSS2 in complex with bacterial toxins (126), antibodies or nanobodies (127, 128) or the receptor-binding domain (RBD) of the human coronavirus (HCoV−) HKU1 spike protein (129, 130).

#### Increasing specificity

Structure-based design should yield compounds that supersede presently available ones in terms of efficacy and specificity (94, 95), and a recent study demonstrated that molecular dynamics simulations jointly with active learning hold potential for inhibitor identification (131). Nevertheless, repurposing of available compounds and improved derivatives remains a viable strategy (132). Nanobody-based approaches targeting the protease domain of TMPRSS2 should allow for highly specific inhibition (133). In this context, it will be interesting to determine whether targeting not only the protease domain but also the SCRC interferes with substrate recognition, since the SCRC might ensure the correct spatial orientation of the protease domain.

#### Interference with TMPRSS2 expression and exploitation of endogenous inhibitors

Targeting TMPRSS2 for degradation, for instance via proteolysis-targeting chimeras (PROTACs) (134), is a promising approach to exploit this enzyme for antiviral therapy (135). Similarly, anti-androgen drugs can reduce TMPRSS2 expression and may have protective effects in the context of COVID-19 (136, 137), although unambiguous results from clinical studies are currently missing. Host-encoded protease inhibitors can be exploited for the inhibition of coronavirus infection (138). For instance, Trypstatin inhibits TMPRSS2 with similar efficiency as Camostat and was found to block cell entry of diverse coronaviruses (139). Further, Trypstatin reduced viral titers and clinical signs in a hamster model of SARS-CoV-2 infection (139). Similarly, α1-antitrypsin (α1AT), which is present at high levels in the circulation, inhibits TMPRSS2 activity and SARS-CoV-2 infection of lung cells (140) and deserves further exploration. Finally, antiviral efficacy can be increased by simultaneously targeting TMPRSS2 and other cellular (141) or viral proteases (142) required for infection.

All approaches discussed above should consider potential cell type–specific differences in glycoprotein cleavage as well as the subcellular localization of glycoprotein processing by TMPRSS2 since priming of IAV hemagglutinin occurs intracellularly (143) and is particularly efficient in ciliated epithelial cells (144).

## RECENT DEVELOPMENTS

In the last section of this review, we will focus on recent developments with significance to antiviral intervention—the finding that TMPRSS2 can serve as a coronavirus receptor and not just as activating protease, and a study shedding new light on the TMPRSS2 dependence of Omicron subvariants.

### TMPRSS2—a HKU1 receptor

ACE2, DPP4, CD13, and DPEP1, structurally related cell surface proteases involved in peptide metabolism, are receptors used by alpha- and beta- and deltacoronaviruses for cell entry and receptor choice is believed to play a central role in viral cell, organ, and species tropism. Recent studies suggest that TMPRSS2 joins this prominent list—the protease was identified as a receptor for the human coronavirus HKU1 (133). A catalytically inactive TMPRSS2 mutant unable to cleave the HKU1 spike protein was nevertheless able to bind to the HKU1 spike and support HKU1 entry (133). Furthermore, conserved residues in the HKU1 RBD were required for TMRSS2 binding and nanobodies directed against TMPRSS2 inhibited cell entry of HKU1. Collectively, these findings define TMPRSS2 as a bona fide receptor for HKU1 and not only an activating protease.

**Fig 4:**
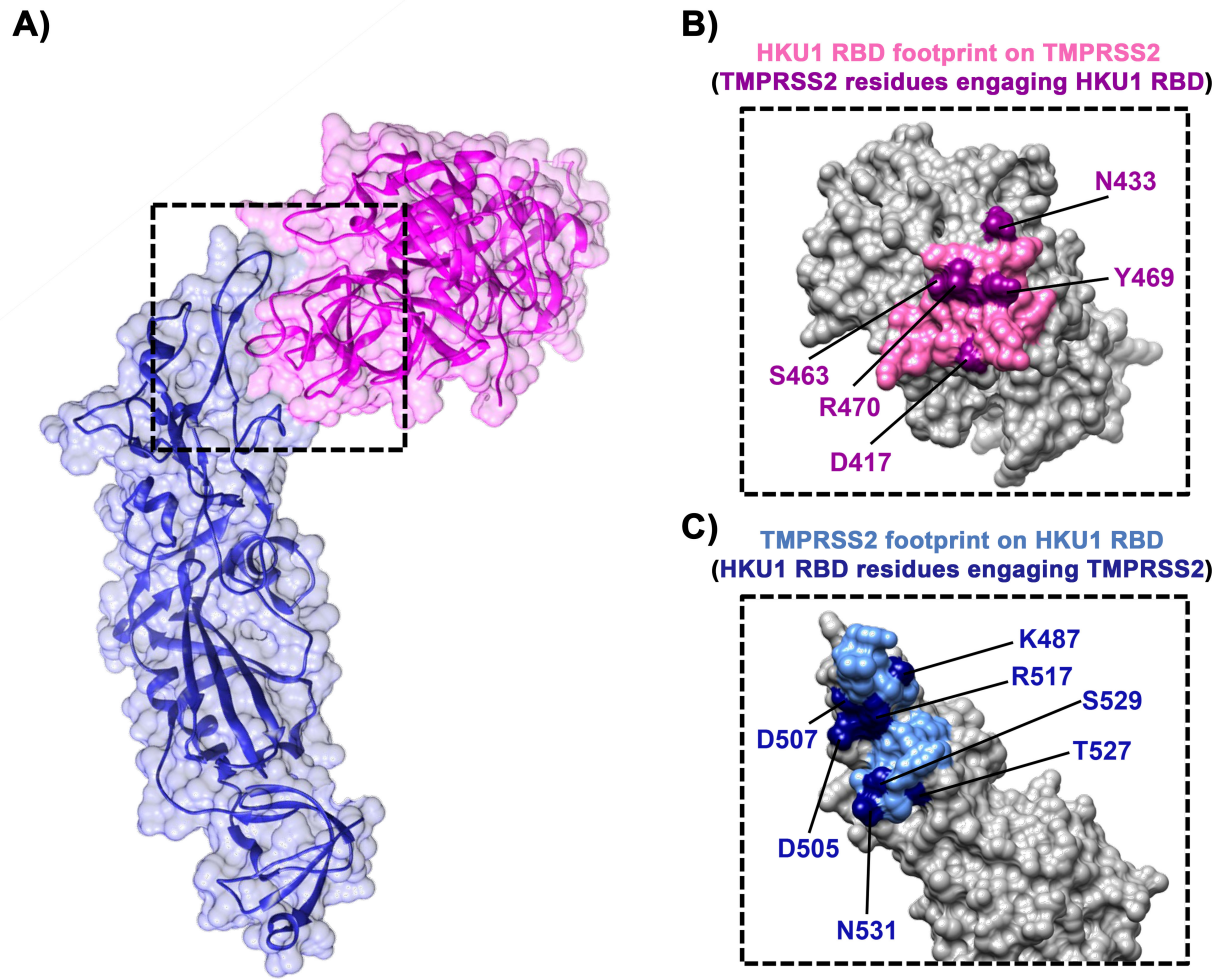
TMPRSS2 is the cellular receptor for human coronavirus HKU1. (**A**) Protein structure of the receptor-binding domain (RBD) of human coronavirus HKU1 (blue) bound to the protease domain of TMPRSS2 (pink). Models are based on the crystal structure PDB:8S0M (128). (**B**) Close-up on the TMPRSS2/HKU1-RBD interface in which the HKU1-RBD footprint on the TMPRSS2 protease domain is indicated (pink) and TMPRSS2 residues that directly interact with HKU1-RBD are highlighted (purple). (**C**) Close-up on the TMPRSS2/HKU1-RBD interface in which the TMPRSS2 footprint on the HKU1-RBD is indicated (light blue) and HKU1-RBD residues that directly interact with TMPRSS2 are highlighted (dark blue).

Subsequent structural and functional studies provided mechanistic insights (145) (Fig. 4): the RBDs within the HKU1 spike protein trimer are typically present in a down conformation, which limits exposure to the immune system. Binding of the N-terminal domain (NTD) of HKU1 spike to cellular sialoglycans initiates the transition of the RBD into the up conformation (128, 129, 146, 147), which is required for binding to TMPRSS2 (127–130) but also exposes epitopes for neutralizing antibodies. The receptor-binding motif (RBM) of the HKU1 RBD binds closely to the catalytic site of TMPRSS2 but does not make atomic contacts, resulting in inhibition of ligand processing by TMPRSS2 and explaining why an inactive TMPRSS2 mutant can still serve as receptor (148). Finally, transition from the zymogen to the enzymatically active form results in conformational changes that favor recognition of ligands and increase binding to the HKU1 RBD (128). In sum, the adaptation of HKU1 to usage of TMPRSS2 as receptor does not obviate the need for an activating protease but provides the viral spike protein with a docking surface for cell entry. However, the conformational changes required for receptor binding increase the vulnerability of spike to neutralizing antibodies and therefore occur only upon successful attachment of virions to the cell surface, thereby limiting exposure to antibodies and promoting sustained circulation of the virus in the human population.

### ACE2-TMPRSS2 interactions—determinants of TMPRSS2 usage?

Pre-Omicron variants (Alpha, Beta, Gamma, and Delta) exhibit strong dependence on TMPRSS2 for entry into lung epithelial cells, thereby circumventing endosomal restriction factors, including interferon-induced transmembrane (IFITM) proteins (149–152), that can limit cathepsin L-dependent entry, although the role of IFITM proteins in SARS-CoV-2 entry remains debated (153). In contrast, most Omicron subvariants display reduced S1/S2 cleavage (44, 154, 155) and diminished TMPRSS2 usage, favoring cathepsin L-dependent endosomal entry *in vitro* (43–45), a shift that correlates with impaired infection of lung-derived cell cultures and reduced lower-respiratory-tract pathogenicity in patients (156). The efficient lung cell entry of BA.5 despite inefficient TMPRSS2 usage (157), and the reacquisition of both robust TMPRSS2 usage and efficient lung cell entry by BA.2.86 (158, 159) constitute notable exceptions and indicate that SARS-CoV-2 can reacquire traits associated with high virulence that were presumed lost during variant evolution. In contrast, murine infection models revealed that Omicron variant dissemination in the airways remains largely TMPRSS2 dependent (64, 65). This observation underscores the limitations of cell-line systems and emphasizes that the determinants governing TMPRSS2 usage by pre-Omicron variants and Omicron subvariants remain incompletely understood.

A recent preprint suggests that the physiological functions of ACE2 and their consequences for ACE2-TMPRSS2 interactions may help explain the altered TMPRSS2 dependence of Omicron subvariants (160). ACE2 is a zinc-dependent carboxypeptidase of the renin-angiotensin system (RAS) that converts angiotensin II to angiotensin-(1-7) and exerts protective effects against lung injury (161). In addition, ACE2 functions as a structural chaperone for the amino/imino acid transporters SLC6A19 (B0AT1) and SLC6A20 (SIT1/IMINO), both of which require ACE2 for proper trafficking and surface expression and function (162). The study reports that ACE2 directly interacts with TMPRSS2 and that formation of ACE2-TMPRSS2 complexes is incompatible with efficient infection by Omicron subvariants. Notably, the observed reduction in infection was not attributable to impaired viral entry in a single-cycle assay but correlated with diminished cleavage at the S1/S2 site and thus reduced multicycle replication. Coexpression of SLC6A19 or SLC6A20 disrupted ACE2-TMPRSS2 complex formation, restoring efficient infection by Omicron subvariants. Collectively, these findings suggest that pre-Omicron variants can exploit ACE2-TMPRSS2 complexes for infection, whereas Omicron subvariants require disruption of these complexes, either through ACE2 mutations that alter TMPRSS2 binding and TMPRSS2-mediated ACE2 processing (163) or through competitive binding of ACE2 by its physiological transporter partners. On a more general level, TMPRSS2 usage by coronaviruses might depend on cellular factors that modulate TMPRSS2 interactions with their respective cellular receptors—a conclusion supported by the finding that CD9 promotes formation of DPP4-TMPRSS2 complexes and thereby augments MERS-CoV entry (164).

## CONCLUSIONS

TMPRSS2 is an attractive target for antiviral intervention in IAV and coronavirus infection. Disappointing results obtained in clinical trials with repurposed protease inhibitors likely reflect insufficient drug concentrations in the respiratory tract; topical application may be key to resolving this issue, and initial studies with Aprotinin demonstrate the high potential of this approach. Further optimization of inhibitors and the use of innovative delivery systems are expected to improve efficacy. To determine the spectrum of antiviral activity and the potential of TMPRSS2 inhibitors for pandemic preparedness, it will be important to assess whether the TMPRSS2 dependence observed in cell culture for respiratory viruses beyond those discussed here extends to the infected host. In addition, further research into the role of TMPRSS2 in proinflammatory cytokine and chemokine responses is needed to fully evaluate the therapeutic potential of TMPRSS2 inhibitors. Finally, the discovery that TMPRSS2 functions as a bona fide receptor for the human coronavirus HKU1 highlights the importance of continued research into the fundamental roles of TMPRSS2 in viral spread and pathogenesis.
